# Supplementary material for: Revealing the Immune Heterogeneity between Systemic Lupus Erythematosus and Rheumatoid Arthritis Based on Multi-Omics Data Analysis
Source: Int J Mol Sci. 2022 May 5;23(9):5166. doi: 10.3390/ijms23095166 (PMC9101622; doi:10.3390/ijms23095166)
Supplement: Supplementary file 1 [file ijms-23-05166-s001.zip › Supplementary_tables_caption.pdf]

Supplementary Tables

Table S1: Each TRBV and TRBJ gene's frequency in each SLE, RA and HC sample. Row 2-19 represents the frequency of each TRBV and TRBJ gene in each SLE, RA and HC sample. Row 20-22 represents the numbers of TRBV and TRBJ genes in all the samples of one condition including SLE, RA and HC. Row 23-25 represents the sum of TRBV and TRBJ genes' frequency from all the samples of one condition. Row 26-28 represents the p-value of each TRBV and TRBJ gene's frequency between SLE group, RA group and HC group. The cells with the highlighted red show genes which are differentially expressed between two groups. ($p\text{-value} < 0.05$)

Table S2: Each IGHV and IGHJ gene's frequency in each SLE, RA and HC sample. Row 2-19 represents the frequency of each IGHV and IGHJ gene in each SLE, RA and HC sample. Row 20-22 represents the numbers of IGHV and IGHJ genes in all the samples of one condition including SLE, RA and HC. Row 23-25 represents the sum of IGHV and IGHJ genes' frequency from all the samples of one condition. Row 26-28 represents the p-value of each IGHV and IGHJ gene's frequency between SLE group, RA group and HC group. The cells with the highlighted color red show genes which are differentially expressed between two groups. ($p\text{-value} < 0.05$)

Table S3: Each immune cell type's proportion in each SLE, RA and HC sample.

Table S4: Differentially expressed genes (DEG) in each immune cell type when comparing SLE group to HC group. These DEGs were used for further functional and signaling enrichment analysis.

Table S5: Differentially expressed genes (DEG) in each immune cell type when comparing RA group to HC group. These DEGs were used for further functional and signaling enrichment analysis.
